# Early corticospinal tract sub-pathway lesion load and integrity predict post-stroke motor outcomes

**DOI:** 10.3389/fnhum.2025.1598598

**Published:** 2025-07-01

**Authors:** Xin Wen, Wentao Zeng, Chiyin Li, Yue Qin, Yanqiang Qiao, Tao Lu, Wanghuan Dun, Ming Zhang, Junya Mu

**Affiliations:** ^1^Department of Medical Imaging, The First Affiliated Hospital of Xi’an Jiaotong University, Xi’an, Shaanxi, China; ^2^Xi’an Academy of Fine Arts, Xi’an, Shaanxi, China; ^3^Department of Radiology, Xi’an Daxing Hospital, Xi’an, Shaanxi, China; ^4^Department of Rehabilitation Medicine, The First Affiliated Hospital of Xi’an Jiaotong University, Xi’an, Shaanxi, China

**Keywords:** stroke, motor recovery, corticospinal tract, diffusion spectrum imaging, lesion load

## Abstract

**Introduction:**

Growing evidence suggests that corticospinal tract (CST) damage and microstructural integrity are key predictors of post-stroke motor impairment. However, their combined clinical utility—particularly in CST sub-pathways originating from non-primary motor cortical areas—remains underexplored. This study aimed to determine whether microstructural integrity and lesion load (LL) of each CST sub-pathway at 2 weeks predict motor outcomes at 2, 6, and 12 weeks post-stroke.

**Methods:**

Fifty seven participants completed motor and neuroimaging evaluations at 2 weeks post-stroke and underwent follow-up motor assessments at 6 (*n* = 37) and 12 weeks (*n* = 34). The integrity of the CSTs was quantified using diffusion spectrum imaging (DSI), while CST-LL was measured using structural magnetic resonance imaging, both based on the sensorimotor area tract template atlas. Stepwise multiple linear regression models were used to assess the predictive value of CST microstructural integrity and CST-LL in each sub-pathway at 2 weeks for motor function at 2, 6, and 12 weeks post-stroke.

**Results:**

The results indicated CST integrity and CST-LL were both the main determinants of motor deficit at 2 weeks post-stroke. Specifically, the integrity of CSTs from the primary motor cortex (M1), reflected by fractional anisotropy, emerged as a significant predictor of post-stroke motor deficit at 2 weeks, whereas CST integrity from the dorsal premotor cortex (PMd), reflected by generalized fractional anisotropy, quantitative anisotropy, and radial diffusivity. CST-LL originating from non-M1 motor areas, such as primary sensory cortex (S1), were also the main determinants for motor impairment at 2 weeks post-stroke. However, compared to CST integrity, CST-LL from non-M1 motor areas, including both the PMd and S1, were more dominant predictors, explaining 68.3% (*R*^2^_*adjusted*_ = 0.683, *p* < 0.001) and 79.5% (*R*^2^_*adjusted*_ = 0.795, *p* < 0.001) of the variance in motor outcomes at 6 and 12 weeks.

**Conclusion:**

The microstructural integrity of the PMd tracts and CST-LL from the non-M1 motor areas may be promising biomarker for post-stroke motor impairment. These findings highlight the pivotal role of non-M1 tracts in post-stroke motor function, particularly the PMd tracts, as a potential intervention target to enhance motor recovery.

## 1 Introduction

Motor impairment is the most common functional deficit after stroke (Langhorne et al., 2009), primarily attributed to corticospinal tract (CST) damage. However, accurately predicting post-stroke motor performance remains a considerable challenge (Winters et al., 2015), as clinical factors influencing stroke recovery, including age (Winovich et al., 2017; Alawieh et al., 2018), sex (Roy-O’Reilly and McCullough, 2018), and lesion volume (Liew et al., 2023), fail to fully account for motor outcomes, compounded by inter-individual heterogeneity. This challenge has driven increased exploration of neuroimaging biomarkers for motor recovery, particularly CST damage and microstructural integrity, which may provide valuable insights for personalized rehabilitation (Boyd et al., 2017; Stinear, 2017).

Previous studies have investigated the relationship between CST damage or microstructural integrity and post-stroke motor impairment, as well as the predictive value for motor recovery (Kim and Winstein, 2017; Puig et al., 2017). CST lesion load (CST-LL), which quantifies the overlap between the stroke lesion and CST, reflects CST damage severity. Previous studies have demonstrated that greater CST-LL from the primary motor cortex (M1) in the acute or chronic phase correlates with worse upper extremity motor function post-stroke (Pineiro et al., 2000; Zhu et al., 2010; Feng et al., 2015). Given that approximately 50% of the CST descends from non-M1 motor areas (Seo and Jang, 2013; Welniarz et al., 2017), such as the medial and lateral premotor cortex (Dum and Strick, 1991), recent studies have examined CST-LL in these regions using a fine-grained CST map, highlighting their contribution to post-stroke motor deficit (Riley et al., 2011; Boccuni et al., 2019; Ito et al., 2022; Saltão, Da Silva et al., 2022). However, assessing CST-LL from different cortical origins alone may be insufficient to explain early post-stroke motor impairment.

Diffusion tensor imaging (DTI) is the most commonly used noninvasive method to assess CST microstructural integrity following stroke (Moura et al., 2019). Fractional anisotropy (FA) is the most widely used DTI-derived metric for assessing CST microstructural integrity (Zolkefley et al., 2021). Although most prior studies have primarily focused on the relationship between FA in CST fibers originating from the M1 and post-stroke motor deficit, emerging evidence indicates that this association is not limited to the M1 fibers. A recent study using refined CST templates demonstrating that the FA values of the ipsilesional supplementary motor area (SMA) fibers also correlates with motor outcomes in chronic stroke (Liu et al., 2020), highlighting the potential relevance of non-M1 fibers in motor recovery. However, its limitations in resolving complex fiber architecture result in inaccurate fractional anisotropy (FA) estimates (Volz et al., 2018). Diffusion spectrum imaging (DSI), a higher-order diffusion model, may address the limitations (Wedeen et al., 2005). In addition to standard DTI metrics such as FA, radial diffusivity (RD), mean diffusivity (MD), and axial diffusivity (AD), DSI also provides unique parameters, including quantitative anisotropy (QA), generalized fractional anisotropy (GFA), restricted diffusion imaging (RDI) and isotropic diffusion component (ISO). GFA, the primary DSI-derived metric, characterizes the directional consistency of water diffusion (Basser, 1995). Analogous to FA, lower GFA values indicate greater axonal disruption or loss (Koh et al., 2018). Here, we used different DSI parameters to comprehensively evaluate the microstructural integrity of the CST originating from distinct cortical areas and determine which metrics may effectively represent CST microstructural alterations following stroke.

Although previous studies have explored CST microstructural integrity or CST-LL from different cortical regions, the specific contribution of DSI-derived microstructural integrity and lesion load of CST fibers originating from non-M1 motor areas to motor outcomes in early subacute stroke remains to be fully elucidated.

The primary objectives of this study were (1) to explore which DSI-derived metrics can effectively characterize CST microstructural properties after stroke, (2) to observe the association between microstructural integrity, lesion load of the CSTs from different cortical origins and motor function at 2 weeks post-stroke, and (3) to identify whether microstructural integrity and lesion load within a sub-pathway at 2 weeks following stroke predict motor outcomes at 2, 6, and 12 weeks.

## 2 Materials and methods

### 2.1 Participant selection

This longitudinal study enrolled patients from the Department of Neurology at the Xi’an Daxing Hospital. Inclusion criteria were: (i) first-ever, imaging-confirmed ischemic stroke, (ii) age 18–75 years, (iii) paretic upper extremity (PUE) motor impairment at stroke onset, (iv) right-handedness before stroke, determined by the Edinburgh Handedness Inventory (Oldfield, 1971). Exclusion criteria included: (i) pre-existing neurological or psychiatric disorders, (ii) any contraindications to MRI, (iii) severe persistent aphasia, (iv) PUE orthopedic injuries.

This study was conducted in accordance with the Declaration of Helsinki and was approved by the Institutional Review Board of Xi’an Daxing Hospital (No. Dxll2020-153). Written informed consent was obtained from patients with stroke after a full explanation of the study’s aims and procedures. Age-matched healthy controls were recruited from the Department of Radiology, Xi’an Daxing Hospital, following the same exclusion criteria as stroke participants. All control participants were right-handed. Informed consent was obtained before using their anonymous data for research purposes.

### 2.2 Clinical assessments

Stroke participants underwent motor assessments at 2, 6, and 12 weeks post-stroke, conducted by an occupational therapist. The Fugl–Meyer assessment of the upper extremity (FMA-UE) (Fugl-Meyer et al., 1975) was used to evaluate upper limb motor impairment. This assessment including reflexes, movement and coordination/velocity of PUE consists of 33 items, with a total score range from 0 to 66. Each item is scored on a 3-point ordinal scale: 0 = cannot perform, 1 = performs partially, 2 = performs fully. Lower FMA-UE scores indicate more severe motor impairment.

### 2.3 MRI data acquisition

All participants completed an MRI examination at baseline. Foam cushions and earplugs were used to minimize head movement and scanner noise. During scanning, participants were instructed to stay awake and motionless with their eyes closed. Brain imaging was conducted on a 3T MR scanner (MAGNETOM Prisma, Siemens Healthineers, Erlangen, Germany). The MRI protocols included as follows: (i) Magnetization-Prepared 2 Rapid Acquisition Gradient Echoes (MP2RAGE) (Marques et al., 2010) (repetition time (TR)/echo time (TE)/inversion times (TI1)/TI2 = 5,000/2.98/700/2,500 ms, voxel size = 1.0 × 1.0 × 1.0 mm^3^, field of view (FOV) = 256 × 240 × 176 mm^3^, acquisition time (AT) = 8:20 min); (ii) DSI (TR/TE = 3,300/73 ms, FOV = 220 × 220 × 60 mm^3^, voxel size = 2 × 2 × 2 mm^3^, 128 diffusion direction, b-max = 3,000 sec/mm^2^, AT = 7:22 min); (iii) T2-weighted fluid-attenuated inversion recovery (T2-weighted FLAIR, TR/TE = 9,000/84 ms, FOV = 270 × 320 × 22 mm^2^, voxel size = 0.72 × 0.72 × 6.6 mm^3^, AT = 1:48 min).

### 2.4 Imaging processing

#### 2.4.1 Lesion delineation and image flip

To minimize the impact of high-grade white matter hyperintensities (HWMHs), these were defined as a Fazekas score ≥ 2 in deep and/or periventricular white matter on axial T2-weighted FLAIR using the Fazekas scale (Fazekas et al., 1987). Six patients and 11 controls with HWMHs were excluded.

Stroke lesion masks were manually delineated by an experienced radiologist on uniformed T1-weighted images (UNI) using MRIcron.^1^ Lesion masks were spatially normalized to the Montreal Neurological Institute (MNI) template with Clinical Toolbox in SPM12 (Rorden et al., 2012).

Before analysis, imaging data from 27 stroke participants with right-hemispheric lesions were flipped to the left along the midsagittal line. The normalized lesion masks were also flipped to create an overlap map characterizing the stroke lesion distribution (Figure 2). To balance lesion distribution between hemispheres, the dominant hemispheres of 30 control participants were randomly designated as “affected.”

**FIGURE 1:**
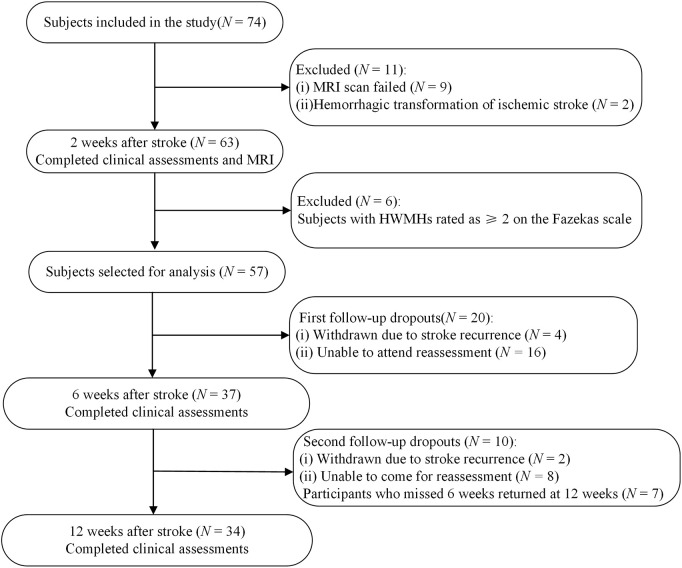
Flowchart of recruitment for the study. MRI, magnetic resonance imaging; HWMHs, high-grade white matter hyperintensities.

**FIGURE 2:**
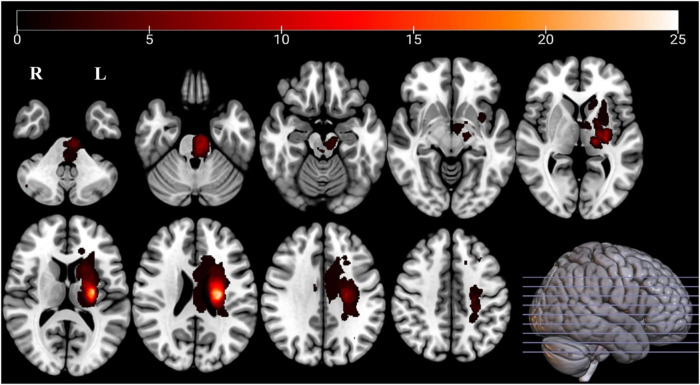
Lesion overlap map of affected voxels across all 57 stroke patients in the left hemisphere. Color bar indicates the number of patients with overlapping lesions. Lesions in the right hemisphere were mirrored to the left. The highest overlap was observed in the internal capsule and brainstem.

#### 2.4.2 DSI Preprocessing and reconstruction

Preprocessing included visual quality control, denoising (Veraart et al., 2016), Gibbs artifact removal (Kellner et al., 2016), susceptibility-induced distortions (Andersson et al., 2003), eddy current-induced distortions and motion correction (Andersson and Sotiropoulos, 2016), and B1 field inhomogeneity bias correction (Tustison et al., 2010), performed using MRtrix3^2^ and FSL 6.0.^3^ Given the absence of a reversed phase-encoding unweighted diffusion image in the MRI protocol, we used the Synb0-DisCo approach to generate a synthetic b0 image without susceptibility-induced distortion from the T1-weighted image (Schilling et al., 2019; Schilling et al., 2020).

DSI data were reconstructed in DSI Studio.^4^ using the q-space diffeomorphic reconstruction (QSDR) approach (Yeh and Tseng, 2011) with a diffusion sampling length ratio of 1.25 and an output resolution of 2 mm. The average values of DSI metrics, including GFA, QA, RDI, ISO, FA, MD, AD and RD, were then calculated for each voxel in the whole brain.

#### 2.4.3 Quantifying lesion load and integrity in CST sub-pathways

To address potential limitations of fiber tracking in stroke-affected brains, we used the sensorimotor area tract template (SMATT) atlas (Archer et al., 2018). SMATT included the CST tracts emanating from six cortical seed regions: M1, ventral and dorsal premotor cortex (PMv and PMd), supplementary and pre-supplementary motor areas (SMA and preSMA), and primary somatosensory cortex (S1). CST-LL for each tract and overall CST was calculated by dividing the number of lesion-overlapping voxels by the total voxel count of the corresponding CST using the SMATT.

Using the SMATT, DSI metrics were extracted for the six CSTs in both ipsilesional and contralesional hemispheres of patients, as well as in both hemispheres of healthy controls. And, the overall CST microstructural integrity was also calculated.

### 2.5 Statistical analysis

All statistical analyses were performed using SPSS 26.0 (IBM Corporation, Armonk, NY, United States). Normality was assessed with the Kolmogorov–Smirnov test and Shapiro–Wilk test. Sex and age differences between stroke and control participants were analyzed using the chi-squared test and independent *t*-tests, respectively.

An independent *t*-test or Mann–Whitney U test was used to compare whole CST microstructural integrity in the ipsilesional hemisphere of stroke patients with that in the corresponding hemisphere of healthy controls. Non-parametric correlation analyses (Spearman’s rho, r_*s*_) were conducted to examine the relationships between ipsilesional CST integrity, subregion-specific CSTs microstructural integrity and CST-LLs from distinct cortical areas, and the FMA-UE scores at 2, 6, and 12 weeks. Significance values were adjusted for multiple comparisons using the false discovery rate (FDR) correction.

Stepwise multiple linear regression models were used to identify independent predictors significantly of PUE motor outcomes at 2, 6, and 12 weeks post-stroke, selecting variables significantly associated with the FMA-UE scores at each time point. Variables were entered at *p* < 0.05 and removed *p* > 0.10. Age, sex, and lesion volume were selected as confounders if significantly correlated with primary outcome. All tests were two-tailed, with *p* < 0.05 considered statistically significant.

## 3 Results

### 3.1 Participant characteristics

Figure 1 depicts the patient selection process for the study. Table 1 summarizes the demographic and clinical characteristics. Fifty-seven stroke patients (56.05 ± 9.73 years, 82.5% male) and 57 healthy controls (55.28 ± 7.87 years, 71.9% male) were included, with no significant differences in age or sex distribution between the groups (*p* > 0.05). A lesion heat map for all 57 patients is shown in Figure 2.

**TABLE 1:** Demographic and clinical characteristics of the patients and controls.

	Patients with stroke (*n* = 57)	Healthy controls (*n* = 57)	t/χ^2^	*p-*value
Age, y	56.05 ± 9.73	55.28 ± 7.87	0.466	0.642
Male, n (%)	47 (82.5%)	41 (71.9%)	1.794	0.180
Left hemispheric lesion, n (%)	30 (54.2%)	–	–	–
Lesion volume, median, IQR (cm^3^)	1.63 (0.64, 3.59)	–	–	–
FMA-UE scores at 2 weeks, median (IQR)	54 (33, 63.5)			
FMA-UE scores at 6 weeks, median (IQR)	64 (48.5, 66)			
FMA-UE scores at 12 weeks, median (IQR)	64.5 (60.5, 66)			

IQR, interquartile range; FMA-UE, Fugl–Meyer assessment of the upper extremity; Values are expressed as the mean ± standard deviation, unless otherwise indicated.

### 3.2 Alterations in CST white matter microstructural integrity following stroke

**FIGURE 3:**
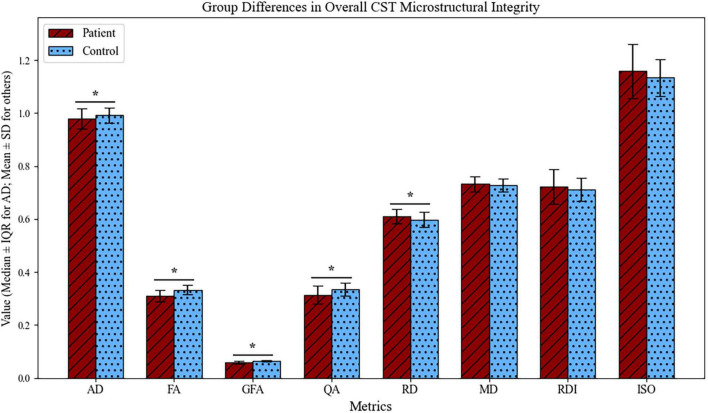
Group differences in CST microstructural integrity metrics between stroke patients and healthy controls (FDR-adjusted). This bar plot illustrates the median and interquartile range (IQR) for AD, as well as the mean and standard deviation (SD) for the other metrics, comparing stroke patients (dark red, hatched) and healthy controls (light blue, dotted). FA, fractional anisotropy; RD, radial diffusivity; MD, mean diffusivity; AD, axial diffusivity; QA, quantitative anisotropy, GFA, generalized fractional anisotropy; RDI, restricted diffusion imaging; ISO, isotropic diffusion component; CST, corticospinal tract. Asterisks (*) denote statistically significant differences after false discovery rate (FDR) correction.

Compared to the healthy controls, CST microstructural integrity, as reflected by AD, FA, GFA, and QA, was reduced, while RD indicated an increase in the stroke group (*p* < 0.05, FDR-adjusted), as shown in Figure 3. In contrast, MD, RDI, and ISO showed no significant group differences (*p* > 0.05, FDR-adjusted).

### 3.3 Associations between CST integrity, lesion load, and motor function post-stroke

MD, RDI, and ISO were excluded from the correlation analysis. Instead, we examined the relationships between PUE motor function at 2 weeks and CST integrity—both overall and from distinct cortical areas—as reflected by AD, GFA, FA, QA, and RD, along with age, sex, and lesion volume. Correlation results for motor outcomes at 6 and 12 weeks post-stroke are provided separately in the Supplementary Material.

**FIGURE 4:**
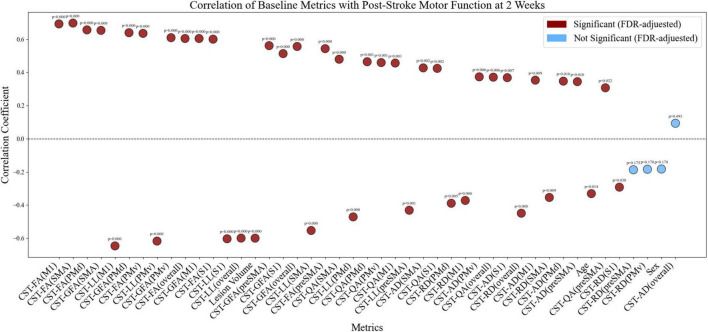
Correlations of CST-LL, CST microstructural integrity, and confounders with PUE motor scores at 2 weeks post-stroke. Dark red dots represent statistically significant correlations after false discovery rate (FDR) correction, while light blue dots indicate non-significant correlations. This figure displays the correlation coefficients between CST-LL, various CST microstructural integrity metrics, confounders and upper limb motor function scores at 2 weeks in stroke patients. The confounders include age, sex, lesion volume. CST, corticospinal tract; LL, lesion load; PUE, paretic upper extremity; FA, fractional anisotropy; RD, radial diffusivity; AD, axial diffusivity; QA, quantitative anisotropy, GFA, generalized fractional anisotropy.

With the exception of sex, the integrity of the preSMA and PMv tracts, reflected by RD, and overall CST integrity reflected by AD, all other CST integrity metrics – including GFA, FA, QA, and AD—were positively correlated with the FMA-UE scores at 2 weeks post-stroke (*p* < 0.05, FDR-adjusted). In contrast, age, lesion volume, overall CST-LL, CST-LLs emanating from diverse motor origins, overall CST integrity, the integrity of the M1, PMd, S1, and SMA tracts reflected by RD were negatively correlated with the FMA-UE scores (*p* < 0.05, FDR-adjusted) (Figure 4). Positive correlations suggest that lower CST integrity is associated with worse PUE motor function, whereas negative correlations indicate that greater CST-LL or microstructural alterations correspond to poorer motor outcomes.

### 3.4 Predictive model for PUE motor function based on CST microstructural integrity and CST-LL

The results of stepwise multiple linear regression analysis for the FMA-UE scores at 2, 6, and 12 weeks post-stroke are presented in Table 2. Each model demonstrated a significant regression equation, with adjusted *R*^2^-value of 0.655 (*p* < 0.001), 0.628 (*p* < 0.001), 0.558 (*p* < 0.001), 0.656 (*p* < 0.001), and 0.525 (*p* < 0.001) for Models 1, 2, 3, 4, and 5, respectively. In Model 1, the integrity of the M1 tracts, as reflected by FA, emerged as a significant predictor of post-stroke motor function. Model 2, 3, and 4 identified CST integrity from the PMd–reflected by GFA, QA, and RD–as a significant contributor to motor outcomes. Model 5 identified CST-LL from the S1 and PMv as key predictors, while CST integrity showed no significant contribution.

**TABLE 2:** Stepwise multiple linear regression models for predicting post-stroke motor function.

Model	1	2	3	4	5	6	7
Follow-up time (week)	2					6	12
CST Integrity Metrics	FA	GFA	QA	RD	AD	FA/GFA/QA/ RD/AD	FA/GFA/QA/ RD/AD
**Predictive variables**	β_std_	β_std_	β_std_	β_std_	β_std_	β_std_	β_std_
CST integrity (PMd)		0.443***	0.369***	–0.376***			
CST Integrity (M1)	0.623***						
CST- LL (PMd)						–0.392**	–0.336***
CST- LL (PMv)				–0.321***	–0.443**		
CST- LL (S1)		–0.455***	–0.616***	–0.478**	–0.356**	–0.567***	–0.685***
CST-LL (preSMA)	–0.414***						
Age		–0.175*					
R^2^_adj_	0.655	0.628	0.558	0.656	0.525	0.683	0.795

(⋅) indicates the different cortical area of the sub-pathway of CST. CST, corticospinal tract; β_std_, standardized beta coefficients; R^2^_adj_, adjusted R^2^; LL, lesion load; M1, primary motor cortex; PMv and PMd, dorsal and ventral premotor areas; S1, primary somatosensory cortex; SMA and preSMA, supplementary and pre-supplementary motor areas. FA, fractional anisotropy; GFA, generalized fractional anisotropy; QA, quantitative anisotropy; RD, radial diffusivity; AD, axial diffusivity. ***p < 0.001, **p < 0.01, *p < 0.05.

Additionally, in Model 2, older age was linked to poorer motor function. Model 1 identified CST-LL originating from the preSMA as a key predictor. In Model 2, 3, 4, and 5, CST-LL from the S1 was a common predictor, while Model 4 and 5 also identified CST-LL from the PMv as a key predictor. These results indicate that CST-LL from non-M1 motor areas serves as more significant role in predicting motor function at 2 weeks post-stroke than CST-LL from M1. Furthermore, at 6 and 12 weeks post-stroke, CST-LL originating from non-M1 motor areas, including the PMd and S1, emerged as more important predictors than CST integrity. Model 6 accounted for 68.3% (*R*^2^_adjusted_ = 0.683, *p* < 0.001) of the variance in PUE motor outcomes at 6 weeks post-stroke, while Model 7 explained 79.5% (*R*^2^_adjusted_ = 0.795, *p* < 0.001) at 12 weeks (Table 2).

## 4 Discussion

This study examined whether microstructural integrity and CST-LL within sub-pathways at 2 weeks could predict motor outcomes at 2, 6, and 12 weeks post-stroke. CST microstructural integrity and CST-LL in specific sub-pathways, along with age, were identified as significant predictors, of post-stroke motor outcomes at 2 weeks. Notably, CST integrity from the M1, reflected by FA, and from the PMd, reflected by GFA, QA, and RD, served as significant predictors of motor function at 2 weeks post-stroke, highlighting the prognostic value of DSI beyond DTI. CST-LL involving the PMd and S1 tracts emerged as stronger predictors of PUE motor function than CST microstructural integrity at 6 and 12 weeks post-stroke, suggesting that over time, CST-LL becomes a more important determinant of motor outcomes than CST microstructural integrity.

In this study, we used DSI instead of DTI to evaluate changes of CST microstructural integrity. DSI, which provides more detailed diffusion information, enables a more precise characterization of complex white matter fibers and brain microstructure. Our findings showed that AD, FA, GFA, QA values of the ipsilesional CST were significantly lower in stroke patients, while RD values were significantly higher. FA, AD, GFA, and QA values, reflect the diffusion characteristics of water molecules along white matter fibers, with their reduction indicating CST microstructural damage. RD, which directly reflects the properties of myelin sheath, increases in response to myelin damage.

Age has been identified as an independent predictor of motor outcomes following stroke (Stinear, 2017; Alawieh et al., 2018; Jeong et al., 2023). Our findings suggest older individuals, due to greater brain aging, are more prone to severe motor impairment post-stroke. Consistent with previous studies (Zhu et al., 2010; Feng et al., 2015; Ito et al., 2022), lesion volume, although correlated with post-stroke motor outcomes at 2 weeks, did not predict motor deficit at 2, 6 or 12 weeks. This suggests that the impact of CST damage is not masked by lesion volume.

Previous studies have identified CST damage (Pineiro et al., 2000; Zhu et al., 2010; Riley et al., 2011; Feng et al., 2015; Ito et al., 2022) and CST microstructure integrity (Moura et al., 2019) as significant predictors of motor deficit post-stroke. In our study, various combinations of CST microstructural metrics and CST-LL from diverse cortical areas explain 52.5–65.6% of the variability in motor impairment at 2 weeks post-stroke. Similarly, Koyama et al. (2024) reported an explained variance of 63.9% in post-stroke motor outcomes. However, the evaluation of CST-LL and CST microstructural integrity focused on the overall CST rather than specific sub-pathways from distinct cortical areas and relied on DTI for microstructural assessment. Our study found that the microstructural integrity of CST originating from the PMd, quantified by QA, GFA, RD, and from the M1, reflected by FA, serve as important predictors of post-stroke motor function at 2 weeks. This highlights the value of DSI in identifying CST microstructural characteristics from non-M1 motor areas.

The primary sensorimotor cortex (M1 and S1) and the secondary motor cortex (PMd, PMv, preSMA, and SMA) have been identified as key cortical areas for sensorimotor function (Roland and Zilles, 1996; Mayka et al., 2006; Jang, 2014). The secondary motor cortex, also referred to as non-M1 motor areas, includes PMd, PMv, preSMA, and SMA (Chouinard and Paus, 2006). Previous studies have highlighted functional differences among these regions (Rizzolatti and Luppino, 2001; Kantak et al., 2012; Cona and Semenza, 2017) with PMd involved in movement preparation and selection, while PMv plays a role in precise grasping movements.

CST fibers from the M1 descend and cross to the contralateral side, while those from premotor areas connect directly to the ipsilateral side. Despite differences in fiber projections, the number of CST fibers from M1 and the premotor cortex is comparable, and these fibers are anatomically interconnected, functioning in coordination and activating simultaneously during motor tasks (Jang, 2014; Fregosi et al., 2017). Recent studies suggested that, beyond modulating the M1, neurostimulation targeting the PMd may subserve motor recovery, particularly in patients with more severe impairment (Bestmann et al., 2010; Cunningham et al., 2015; Plow et al., 2015; Li J. et al., 2022; Li X. et al., 2022). A previous study (Liu et al., 2020), using a fine-grained template of the CSTs arising from different cortical regions, reported that CST integrity from the M1 and SMA, reflected by FA, is crucial for predicting long-term motor function post-stroke. However, that study did not establish a link between CST integrity from the S1 or premotor cortex and post-storke motor outcomes. Accordingly, our findings further confirm that the CST microstructural integrity from the PMd is equally crucial in predicting motor disorders in the early subacute stroke.

We further explored early predictors of post-stroke PUE motor outcomes at 6 and 12 weeks using stepwise linear regression. The model included the microstructural integrity and lesion load of the CSTs from diverse cortical areas at 2 weeks post-stroke, along with age and lesion volume. Results showed that CST-LL was the sole significant predictor of PUE motor function at 6 and 12 weeks, demonstrating complete dominance over other predictors. CST-LL affecting the PMd and S1 explained 68.3% of the variance in motor outcomes at 6 weeks, and 75.9% at 12 weeks post-stroke. Our findings align with previous studies, confirming that CST-LL in the premotor cortex is a strong predictor of post-stroke PUE motor outcomes, aiding in classifying motor recovery and assessing stroke severity.

We also found that CST-LL originating from the S1 remained a significant predictor of motor function at 2, 6, and 12 weeks post-stroke. Considering that the S1 primarily processes sensory information and integrates information from M1(Umeda et al., 2019), damage to the CST emanating from the S1 may disrupt sensory feedback necessary for motor control, thereby exacerbating motor deficit and hindering functional recovery. Recent studies suggest that neurostimulation targeting the S1 may enhance post-stroke motor recovery (Veldman et al., 2014; Borich et al., 2015; de Freitas Zanona et al., 2022).

This study has several limitations. First, the sample size was small. Future studies should include more participants with moderate-to-severe stroke to validate the findings. Second, lesion heterogeneity across patients may have introduced bias. However, stroke inherently presents with motor deficit from diverse lesion locations. To comprehensively assess CST microstructural integrity and lesion load, we included patients with basal ganglia and brainstem lesions. Finally, relying solely on the FMA-UE scores may not fully capture the complexity of motor function. Future research should incorporate assessments of fine motor skills to better understand the relationship between CST damage from distinct cortical areas and post-stroke motor recovery.

## 5 Conclusion

Using DSI, we demonstrate that the distinct contributions of CST sub-pathway integrity from different motor cortical regions to post-stroke motor function. While the integrity of the M1 sub-pathway fibers was linked to motor impairment, the PMd fiber integrity also showed an additional association, further supporting the role of non-M1 sub-pathway integrity in post-stroke motor function. Over time, lesion load involving the non-M1 fibers becomes an increasingly dominant predictor of motor outcomes.

In conclusion, our study provides further evidence that CST sub-pathways from non-M1 motor areas, particularly the PMd and S1, serve as a vital role in post-stroke motor recovery. These non-M1 fibers, with flexible functional roles, are valuable for assessing motor deficit post-stroke and guiding rehabilitation strategies.

## Data Availability

The original contributions presented in this study are included in this article/Supplementary material, further inquiries can be directed to the corresponding authors.

## Footnotes

1
www.nitrc.org/projects/mricron

2
https://www.mrtrix.org/

3
www.fmrib.ox.ac.uk/fsl

4
https://dsi-studio.labsolver.org
