# Correction: Early corticospinal tract sub-pathway lesion load and integrity predict post-stroke motor outcomes

**DOI:** 10.3389/fnhum.2025.1667293

**Published:** 2025-07-29

**Authors:** Xin Wen, Wentao Zeng, Chiyin Li, Yue Qin, Yanqiang Qiao, Tao Lu, Wanghuan Dun, Ming Zhang, Junya Mu

**Affiliations:** ^1^Department of Medical Imaging, The First Affiliated Hospital of Xi'an Jiaotong University, Xi'an, Shaanxi, China; ^2^Xi'an Academy of Fine Arts, Xi'an, Shaanxi, China; ^3^Department of Radiology, Xi'an Daxing Hospital, Xi'an, Shaanxi, China; ^4^Department of Rehabilitation Medicine, The First Affiliated Hospital of Xi'an Jiaotong University, Xi'an, Shaanxi, China

**Keywords:** stroke, motor recovery, corticospinal tract, diffusion spectrum imaging, lesion load

In the published article, there was an error in the legend for Table 1 as published. The legend incorrectly included the abbreviations “MMSE, Mini-Mental State Examination; NIHSS, National Institute of Health Stroke Scale,” which should have been removed because the table does not contain these variables.

The corrected legend appears below.

IQR, interquartile range; FMA-UE, Fugl–Meyer assessment of the upper extremity; Values are expressed as the mean ± standard deviation, unless otherwise indicated.

In the published article, there was an error in the figure and caption for Figure 1 as published. Due to a revision based on the reviewers' suggestion to remove the NIHSS assessment, the NIHSS label was inadvertently retained in the flowchart and should have been deleted. In addition, to ensure consistency in terminology, the label “FMA-UE” was also replaced with “clinical assessments” in the flowchart. The corrected figure appears below.

**Figure 1:**
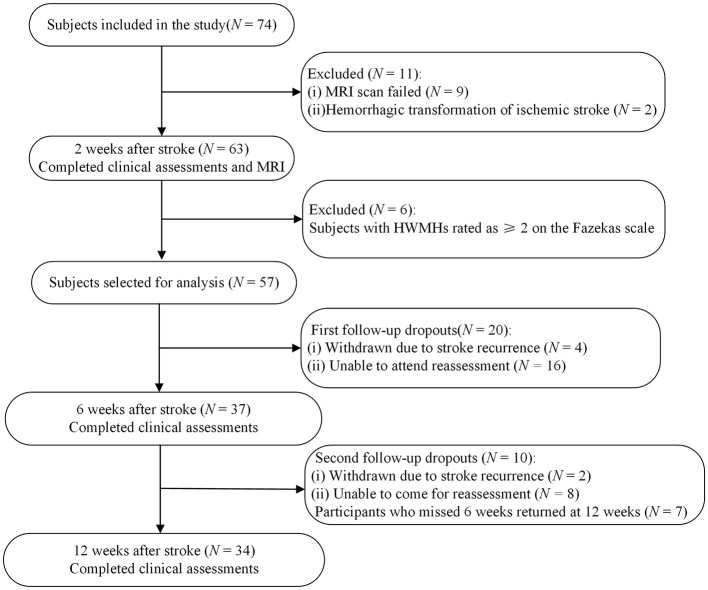
Flowchart of recruitment for the study. MRI, magnetic resonance imaging; HWMHs, high-grade white matter hyperintensities.

The original article has been updated.

