# Supplementary material for: Early corticospinal tract sub-pathway lesion load and integrity predict post-stroke motor outcomes
Source: Front Hum Neurosci. 2025 Jul 1;19:1598598. doi: 10.3389/fnhum.2025.1598598 (PMC12261457; doi:10.3389/fnhum.2025.1598598)
Supplement: Supplementary file 1 [file Presentation_1.ppt]

## Slide 1
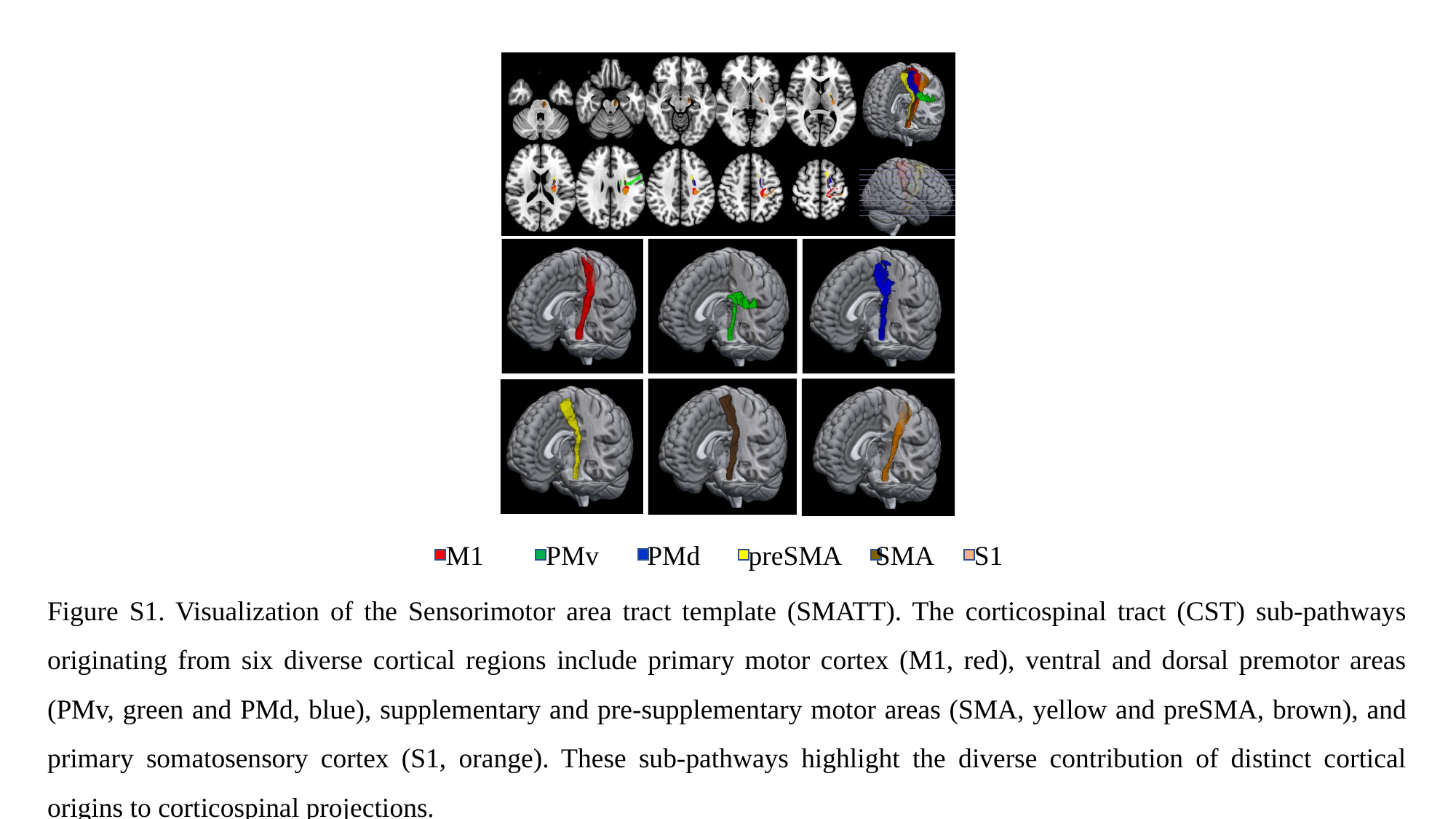

M1 PMv PMd preSMA SMA S1
Figure S1. Visualization of the Sensorimotor area tract template (SMATT). The corticospinal tract (CST) sub-pathways originating from six diverse cortical regions include primary motor cortex (M1, red), ventral and dorsal premotor areas (PMv, green and PMd, blue), supplementary and pre-supplementary motor areas (SMA, yellow and preSMA, brown), and primary somatosensory cortex (S1, orange). These sub-pathways highlight the diverse contribution of distinct cortical origins to corticospinal projections.

## Slide 2
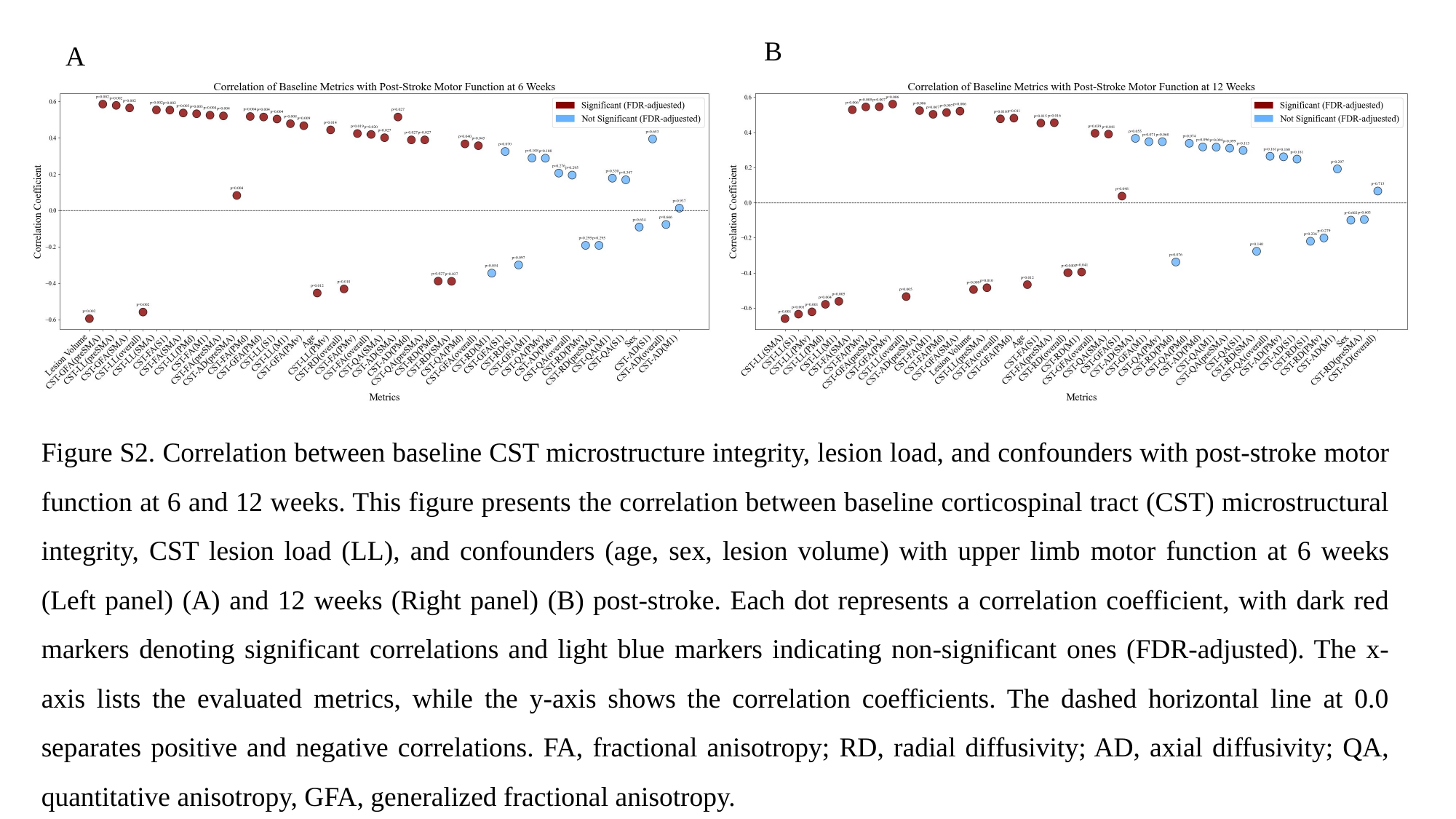

B
A
Figure S2. Correlation between baseline CST microstructure integrity, lesion load, and confounders with post-stroke motor function at 6 and 12 weeks. This figure presents the correlation between baseline corticospinal tract (CST) microstructural integrity, CST lesion load (LL), and confounders (age, sex, lesion volume) with upper limb motor function at 6 weeks (Left panel) (A) and 12 weeks (Right panel) (B) post-stroke. Each dot represents a correlation coefficient, with dark red markers denoting significant correlations and light blue markers indicating non-significant ones (FDR-adjusted). The x-axis lists the evaluated metrics, while the y-axis shows the correlation coefficients. The dashed horizontal line at 0.0 separates positive and negative correlations. FA, fractional anisotropy; RD, radial diffusivity; AD, axial diffusivity; QA, quantitative anisotropy, GFA, generalized fractional anisotropy.
