# Supplementary figures and images for: Early corticospinal tract sub-pathway lesion load and integrity predict post-stroke motor outcomes
Source: Front Hum Neurosci. 2025 Jul 1;19:1598598. doi: 10.3389/fnhum.2025.1598598 (PMC12261457; doi:10.3389/fnhum.2025.1598598)

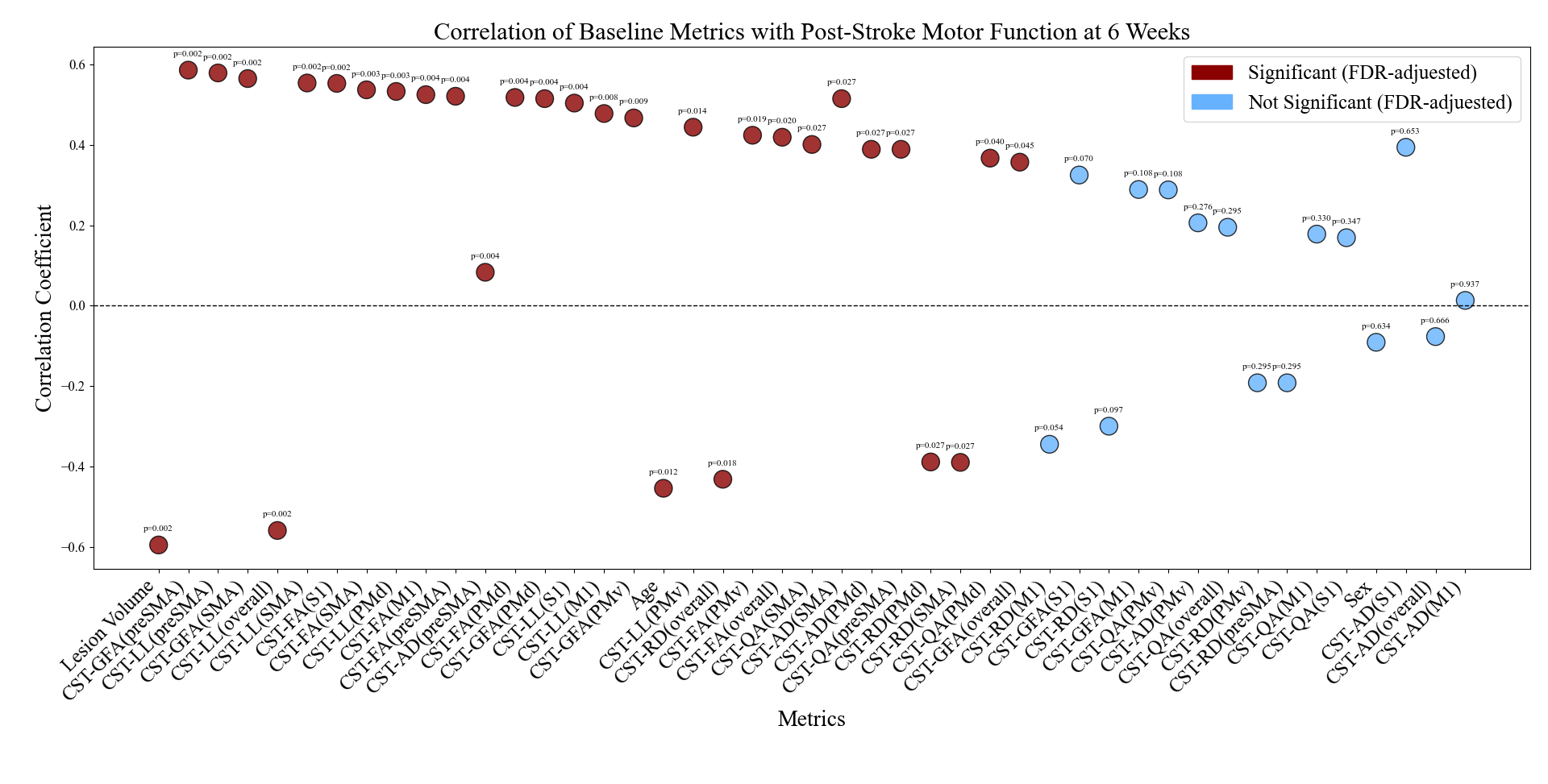

Supplement: Supplementary file 2 [file Image_1.tif]

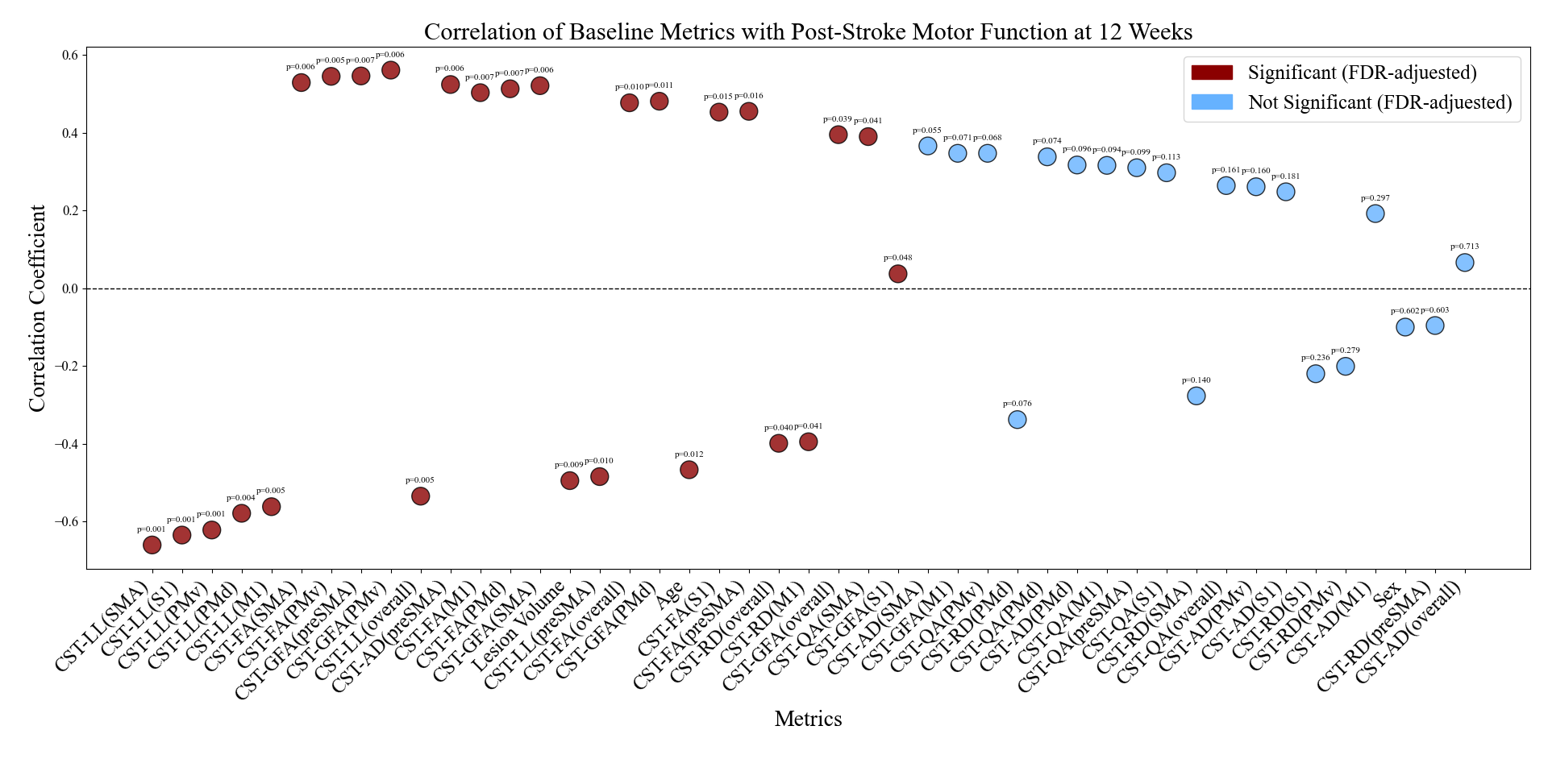

Supplement: Supplementary file 3 [file Image_2.tif]
